# The “Fat but Fit” Paradigm and Bone Health in Young Adults: A Cluster Analysis

**DOI:** 10.3390/nu13020518

**Published:** 2021-02-05

**Authors:** Ana Torres-Costoso, Miriam Garrido-Miguel, Luis Gracia-Marco, Purificación López-Muñoz, Sara Reina-Gutiérrez, Sergio Núñez de Arenas-Arroyo, Vicente Martínez-Vizcaíno

**Affiliations:** 1 Faculty of Physiotherapy and Nursing, University of Castilla La Mancha, 45071 Toledo, Spain; anaisabel.torres@uclm.es (A.T.-C.); purificacion.lopez@uclm.es (P.L.-M.); 2 Health and Social Research Center, University of Castilla-La Mancha, 16071 Cuenca, Spain; sara.reina@uclm.es (S.R.-G.); sergio.nunezdearenas@uclm.es (S.N.d.A.-A.); vicente.martinez@uclm.es (V.M.-V.); 3 Faculty of Nursing, University of Castilla-La Mancha, 16002 Albacete, Spain; 4 PROFITH “PROmoting FITness and Health Through Physical Activity” Research Group, Sport and Health University Research Institute (iMUDS), Department of Physical Education and Sports, Faculty of Sport Sciences, Universidad de Granada, 18071 Granada, Spain; lgracia@ugr.es; 5 Instituto de Investigación Biosanitaria ibs, 18014 Granada, Spain; 6 Facultad de Ciencias de la Salud, Universidad Autónoma de Chile, Talca 3467987, Chile

**Keywords:** bone, body composition, nutrients, fitness, muscular strength, college students

## Abstract

The fat but fit paradox has suggested that obese individuals with good fitness levels have lower cardiometabolic risk compared to individuals with normal weight but lower fitness levels. This paradigm has not been explored in the context of bone health. The aim of this study was to test whether categories of fat but fit paradigm assessed by body fat percentage and handgrip strength holds up in young adults and to analyze the relationship between fat but fit categories and bone outcomes. Cluster cross-sectional analyses of data from 499 young adults aged 18 to 30 from Toledo and Cuenca, Spain were conducted. Body fat percentage, handgrip strength, bone mineral content (BMC), bone mineral density (BMD), and dietary nutrients such as, proteins, magnesium, calcium, phosphorus, potassium, and vitamin D were assessed. Cluster analysis of body fat percentage and handgrip z scores resulted in a classification of four clusters that could be interpreted according to Fat Unfit (FU), Unfat Unfit (UU), Fat Fit (FF) and Unfat Fit (UF) categories. ANCOVA models showed that young adults in clusters with higher handgrip strength levels (FF, UF) and with higher key bone nutrients levels (UF) had significantly higher total BMC values than their peers in the UU and FU cluster categories, after controlling for sex, age and height. This study provides two novel conclusions in relation to the fat but fit paradigm: first, it confirms the construct of the four clusters of body fat percentage and handgrip strength, and second, it reinforces the predictive validity of the fat but fit paradigm categories, indicating the positive effect, although it may not just be a causal relationship, of muscular strength and key bone nutrients on counteracting the negative effect of obesity on bone health.

## 1. Introduction

The fat but fit paradox is a theoretical paradigm based on the evidence emerged in the late 1990s suggesting that moderate-to-high levels of cardiorespiratory fitness (CRF) may counteract the adverse influence of obesity on cardiometabolic risk [1,2]. Consequently, individuals who are overweight or obese but have moderate-good fitness levels, appear to be at lower metabolic risk when compare to those who have normal weight but lower fitness levels. To our knowledge, this paradigm has not been explored in the context of bone health.

Bone mass accrual through the 3rd decade of life is considered a strong determinant of osteoporosis risk later in life [3,4,5]. A large amount of evidence supports the benefits of physical activity for bone health among young people [6,7]. Despite this evidence, the time that young people spend engaging in sedentary activities is increasing, which leads to higher adiposity, lower physical fitness levels [8] and presumable consequences for skeletal development [9].

A complex relationship has been described between adiposity and bone development. Numerous studies have proposed that fat mass has a substantial effect on bone, not only due to increased mechanical loading [10] but also due to the metabolic action of bone–related hormones that are secreted or regulated by adipocytes [11]. However, excessive fat mass may negatively influence bone remodeling through the action of inflammatory cytokines that are released from visceral adipocytes [12]. In this sense, after accounting for lean mass, adiposity seems to be a negative predictor of bone mass in youth [10,13]. Moreover, from a nutritional point of view, dietary intake is an important modifiable factor for both fat mass and bone health, accordingly normal bone metabolism requires a sufficient amount of nutrients, such as magnesium, calcium, phosphorus, vitamin D, potassium and proteins [14] also, nutrients such as calcium may play a significant role in decreasing fat mass [15].

With regard to physical fitness, the relationship between aerobic capacity and bone health has been a debatable issue, because while some studies have reported a positive association with bone mass during growth [16] (probably mediated by muscle mass [17]), other authors have reported that only neuromotor fitness (measured by a composite indicator including muscular fitness and speed), but not aerobic capacity, is related to bone health [18]. Conversely, consistent evidence supports a positive influence of muscular strength on bone health [19]. This association is present at anatomical sites that are local and distant from the muscular action [20] and is related not only to powerful muscle contractions and increased osteogenic stimuli in adjacent bone [21] but also to muscle glycogen metabolism at the distal site [22]. Additionally, muscular strength may attenuate the adverse effect of proinflammatory markers associated with adiposity on bone [23].

There is a lack of studies using statistical clustering techniques aimed at reporting specific information in young populations according to their adiposity and/or muscular strength profile. Moreover, the joint role of adiposity and muscular strength on bone health in young adults remains an important and unexplored clinical topic.

Therefore, the aim of this paper was to test the validity of the fat but fit paradigm in young adults from two points of view: (i) assessing whether the four categories of the fat but fit paradigm exist when body fat percentage and muscular strength and CRF variables are analyzed; and (ii) testing whether there are differences among the fat but fit categories in bone health as an indirect concurrent verification of the fat but fit paradigm premises.

## 2. Materials and Methods

### 2.1. The Study Design and Participants

This was a multicenter cross-sectional study aiming to assess assess dietary habits, and cardiovascular risk that occur during university attendance (participants were aged 18–30 years old, and should not have any type of physical or mental disorder). A total of 1330 first-year university students from the University of Castilla La Mancha, Spain, were invited to involve in the study, and 1043 (78.42%) accepted to participate. In this report, we used data from a subsample of 499 university students in which bone variables were measured. The young adults included in the data analysis for this study did not differ in age, sex or parental socioeconomic status from the whole sample of young adults participating in the trial.

The Clinical Research Ethics Committee of The Virgen de la Luz Hospital in Cuenca REG: 2016jPI1116 approved the study protocol, and once participants were informed verbally and in writing, they were asked to sign a consent form as a condition to participate in the study. As there were no participants under the legal age in Spain (younger than 18 years), written informed consent was individually obtained for each participant. The signed informed consent documents were recorded. The Ethics Committee approved the study protocol, including informed consent and permissions documents. The methods used for this research have been previously employed [24,25].

### 2.2. Study Variables

Anthropometry. Stature (cm), and body mass (kg) were measured by using a wall-mounted stadiometer (Seca-222, Vogel & Halke, Hamburg, Germany) and a scale (Seca-770 scale, Vogel & Halke, Hamburg, Germany) respectively. Body mass index (BMI) was calculated as weight in kilograms divided by the square of the height in meters (kg/m^2^), using the means of the two measurements of weight and height. Waist circumference (cm) was measured at the end of exhalation in the middle point between the costal margin and iliac crest.

Body composition. A dual-energy X-ray absorptiometry (DXA) scanner was used to measure body composition variables (Hologic Discovery Series QDR, Bedford, MA, USA in Toledo and Lunar iDXA, GE Medical Systems Lunar, Madison, WI 53718, USA in Cuenca). In Toledo, the DXA equipment was calibrated by a lumbar spine phantom following the Hologic guidelines. All the DXA scans were analyzed using Physician’s Viewer, APEX System Software Version 3.1.2. (Bedford, MA, USA). In Cuenca, the analyses were performed using enCoreTM 2008 software version 12.30.008. DXA equipment accuracy was checked daily before each scanning session using the GE Lunar calibration phantom, as recommended by the manufacturer. Participants were scanned in the supine position in the middle of the platform A trained researcher performed all scans at high resolution, following the same protocol. Body fat percentage calculated as total fat mass divided by weight, total lean mass (kg), total bone mineral content (BMC) (which is the amount of bone mineral in a specific area, measured in g) and total areal bone mineral density (aBMD) (which is the bone mineral content divided by the bone scanned area, measured in g/cm^2^ thus, conceptually it is the ratio of BMC to bone size) were calculated for each individual from the whole-body scan. After that, as two different DXA devices were used to measure body composition variables, in order to achieve measurement ready to be included in a single analysis, z scores according to the device were calculated and used in all analyses, thus controlling the variability due to the measurement device.

Physical fitness variables were assessed after a 4-min warm-up consisting in calisthenic exercises and static stretching, and included the following:

Muscular strength: The handgrip test was used to measure upper body strength using a TKK 5401 Grip- DW digital dynamometer with adjustable grip (Takeya, Tokyo, Japan). The average of four measurements (two with the right hand and two with the left hand) was reported in kilograms.

Cardiorespiratory fitness (CRF): The Course Navette test (20-m shuttle run test) was evaluated. The participants were asked to run between two lines set 20 m apart by following the pace of the audio signals produced from a CD player. The starting speed was 8.5 km∙h^−1^ and was increased by 0.5 km∙h^−1^ each minute. The participants were equally encouraged to continue the test until they reached maximal effort. The test finished when the participants stopped due to fatigue or when they failed to reach the line two successive time. 

Nutrients: The Food-Frequency Questionnaire (FFQ) [26] was used to determine the total consumption of proteins, calcium, magnesium, phosphorus, potassium, and vitamin D. This validated questionnaire with 9 levels of intake frequencies (never or almost never, between 1 and 3 times per month, once per week, 2–4 times per week, 5–6 times per week, once per day, 2–3 times per day, 4–6 times per day, and more than 6 times per day), included 137 auto reported items for consumption frequency over last year. Spanish food composition tables [27] were used to compute nutrient and energy intakes. 

### 2.3. Statistical Analysis

The normality of the distribution of continuous variables was analyzed using both statistical and graphical procedures using Kolmogorov–Smirnov test and normal probability plots, respectively. In order to show the relationships among variables, and considering that age and sex are covariates that influence most of the variables included in our models, we calculated partial correlation coefficients controlling for age and sex among body composition variables (body fat percentage, total lean mass, total BMC and total aBMD), physical fitness parameters (CRF and handgrip strength), and nutrients (calcium, magnesium, phosphorus, potassium, vitamin D and proteins) were calculated.

**Figure 1:**
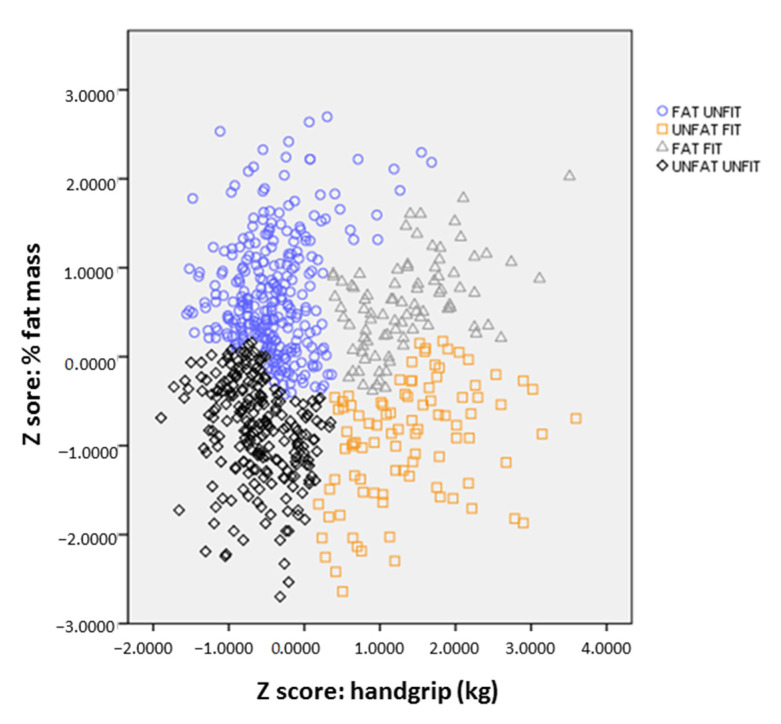
Clustering of individuals according to their body fat percentage and muscular strength (handgrip strength) z scores using the Ward method.

To identify homogenous groups according to the participants’ body composition and physical fitness, based on the z scores of body fat percentage and handgrip strength, a hierarchical cluster analysis was conducted, which no prior information about the group or cluster membership for any of the individuals, using the Ward’s method, based on a squared Euclidean distance [28], clusters individuals into a pre-determined number of groups according to the similarity of the values of the selected variables. Because outliers in cluster analysis are recognized as observations belonging to none of the clusters, clusters procedures can be substantially influenced by few outliers; thus, values of more than three standard deviations (+3 SD) above or below the mean were removed (three young adults) before the analysis [29]. Finally, we included afour-cluster solution with the following categories: (i) Fat Unfit (FU), (ii) Unfat Unfit (UU), (iii) Fat but Fit (FF), and (iv) Unfat Fit (UF) (Figure 1).

Subsequently, ANCOVA models tested mean differences in body composition variables, including BMC and aBMD, physical fitness variables, and nutrients related to bone mass. These variables were used as dependent variables in the assessment of the relationship between fat but fit categories and sex (fixed factors) controlling for height, age and sex. Pairwise post hoc multiple comparisons were examined using the Bonferroni test. Finally, a sensitivity analysis was conducted including body fat percentage and CRF levels z scores as cluster variables (Appendix A). The methodology for this cluster analysis was the same as described above.

Statistical analyses were performed using IBM SPSS Statistics v.24 0 (IBM Corp., Armonk, NY, USA). Statistical significance was set at 0.05.

## 3. Results

Table 1 shows the descriptive characteristics (means ± SDs) of the total study sample by sex. There were statistically significant differences between male and female in weight, BMI, total lean mass, total BMC, total BMD, CRF and handgrip strength

Partial correlation coefficients between total lean mass, CRF, handgrip strength, total BMC and total aBMD after controlling for age and sex are shown in Table 2. Body fat percentage was negatively correlated with CRF, handgrip strength, calcium, magnesium, phosphorus, potassium, and proteins (coefficients range r: −0.668 to −0.146), and positively correlated with total lean mass, BMC and aBMD (coefficients range r: 0.095 to 0.157). Handgrip strength was positively correlated with total lean mass, CRF, BMC and aBMD (coefficients range r: 0.533 to 0.837). Additionally, CRF was positively correlated with total lean mass, handgrip strength, BMC, aBMD, magnesium, phosphorus, potassium, proteins (coefficients range r: 0.149 to 0.633).

Figure 1 shows the four clusters solution that, according to body fat percentage and muscular strength (handgrip) z scores, correspond with the following categories: FU, UU, FF and UF. The ANCOVA models (Table 3) show the mean adjusted differences in body composition and physical fitness variables by the four categories of the cluster solution in terms of muscular strength (handgrip) and body fat percentage. The four cluster solutions show (i) lowest handgrip strength levels and the highest body fat percentage values for the ”FU” category (*n* = 193); (ii) the low values of handgrip strength and standard body fat percentage values for the ”UU” category (*n* = 53); (iii) high levels of handgrip strength and body fat percentage values for the ”FF” category (*n* = 72); and (iv) the highest handgrip strength levels and the lowest body fat percentage values for the ”UF” category (*n* = 78).

The four categories of the cluster in terms of handgrip strength and adiposity variables empirically fit the fat but fit paradigm premises, such that an increasing trend can be observed by cluster category (UU < FU < FF < UF) in terms of muscular strength levels thus, individuals in FF and in UF clusters could be considered individuals with acceptable-to-good levels of muscular strength and, conversely, a decreasing trend (UF > FF > UU > FU) in terms of adiposity variables, although not all post hoc pairwise comparisons achieved statistical significance (*p* < 0.05).

With respect to the bone health differences between fat but fit categories, young adults belonging to clusters with higher handgrip strength levels (FF, UF) had significantly higher total BMC and aBMD than their peers in UU and FU cluster categories, after controlling for sex, age and height.

In relation to the level of nutrients, young adults belonging to clusters with higher handgrip strength and lower body fat percentage levels (UF) had significantly higher levels in the bone related nutrients than their peers, especially those who belong to FU cluster category.

**Figure A1:**
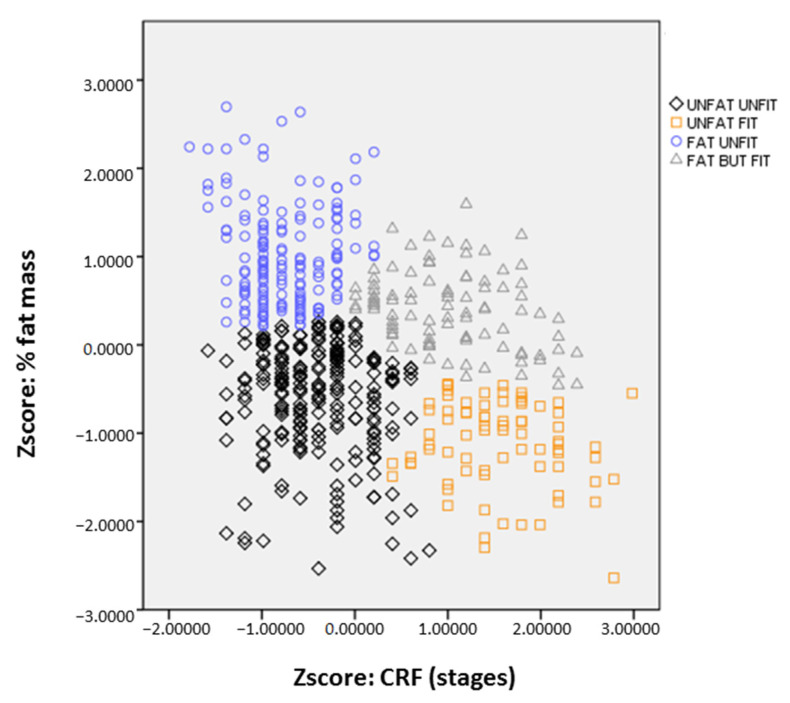
Clustering of individuals according to their body fat percentage and cardiorespiratory fitness (CRF) z scores using the Ward method.

The results were similar in terms of body composition, physical fitness, and dietary nutrients when we classified the individuals according to their body fat percentage and CRF (Appendix Figure A1, Table A1).

## 4. Discussion

This study provides two novel approaches in relation to fat but fit paradigms and their relationship with bone health. First is the empirical confirmation of the construct validity of this theoretical paradigm using statistical clustering techniques to classify young adults according to their body fat percentage and muscular strength (and CRF in a sensitivity analysis) profile. Second, considering the fat but fit hypothesis, as an additional proof of predictive validity, it was determined that fat and unfat individuals with higher levels of fitness showed better bone health and better levels of bone related nutrients than those with lower levels of fitness. Obesity has been increasing in epidemic proportions in children and adults over many decades [30]. Consistent evidence confirms that CRF levels are a major determinant of mortality risk that are able to counteract the negative effects of obesity on cardiovascular risk factors and mortality [31,32]. However, the evidence supporting the validity of this obesity paradox has always been tested using CRF as a fitness indicator, such that the fat but fit paradigm has never been tested using muscular strength as a fitness indicator [1]. Thus, our cluster approach, as well as our ANCOVA models, confirm the plausibility of the hypothesis, since this analysis clearly differentiated four clusters of young adults according to body fat percentage and muscular strength (handgrip) or CRF level.

Obesity in young people has a negative influence on cardiovascular health, but its association with other outcomes, such as bone health, is controversial. While it is well known that a greater body mass increases bone through its bone piezoelectric effect [33], there are several mechanisms related to obesity that make the bone more fragile. This detrimental effect may be due to several factors, including increased production of proinflammatory cytokines leading to the activation of bone-resorbing osteoclasts, excessive secretion of leptin or reduced calcium absorption associated with high fat intake, among others [34].

Our study demonstrates that, in the case of bone health, the fat but fit paradigm is supported in terms of both BMC and aBMD since the influence of obesity on bone health is counteracted by acceptable-to-good levels of muscular strength. Thus, young adults with high body fat percentage but also high fitness levels had a significantly better bone outcome than those with low values of fitness levels independent of body fat percentage. Therefore, according to our data, the special role of muscular strength on bone health is likely due to its influence on lean mass as it has been reported that lean mass is a total mediator of the influence of muscular fitness on bone outcomes [35].

Obesity is defined as the presence of excess fat mass that impairs health, but individuals with obesity usually also present an increased amount of lean mass. The increase in lean mass could explain part of the obesity paradox [36], as it could be related with improved muscular strength, a major determinant of bone health in young adults [19]. Along this line, to test the validity of the construct, we used upper body muscular strength, and this variable has shown a stronger relationship with bone health than lower limb muscular strength [20]. Similar to muscular fitness, our results showed the positive effect of CRF on bone health, probably due to its relationship on the decrease in fat mass, as fat mass has been showed as a mediator in the association between CRF and bone [37].

Our data also display that fat and unfat but fit individuals had a good bone health probably because the effect of obesity on bone health is counteracted not only by good levels of fitness but also by higher levels of key bone nutrients. In this sense, different mechanisms have been proposed to clarify the anti-obesity effect of dietary calcium through its role in the regulation of adipocyte lipid metabolism and triglyceride storage [38] but also, by its effect on gastrointestinal tract leading to increased fecal fat and energy excretion [39].

### Limitations

This study presents some limitations. First, the analysis was cross-sectional; therefore, we cannot make cause-effect inferences. Second, and related to total body bone assessment, there is a consensus that the total body minus the head instead of the total body including the head should be obtained [40] because the skull constitutes a large percentage of the skeleton and is not related to environmental factors. However, most of the studies included only showed data for total body bone measurement. Third, we explored the effect of upper body muscular strength in the fat but fit paradigm, but other fitness components as lower body muscular strength could also impact the bone health of young people. Moreover, different age groups might be explored, but the statistical power was insufficient. Fourth, we tested the effect of body fat percentage to elucidate the obesity paradox and its influences on bone outcomes, but we did not explore other body fat distributions, such as visceral or subcutaneous fat mass. Fifth, the use of self-reported dietary data could result in underreporting or unintentional measurement errors. Sixth, aBMD measured by DXA does not represent the volumetric density but rather the areal density. Seventh, two different DXA devices were used to measure body composition variables, which could bias our results; however, in order to control this source of variability, we have used z scores by device in our analyses. Finally, the relationships among adiposity, fitness and bone health could be potentially confounded by other socio-demographic variables that we did not include in the statistical models.

## 5. Conclusions

In conclusion, our study confirmed the predictive validity of this paradoxical phenomenon by using a cluster analysis to classify young adults according to their body fat percentage and handgrip levels, a different fitness variable from the one usually used to explain the fat but fit paradox, CRF. In addition, the study reinforces the positive effect of upper limb muscular strength and key bone nutrients on counteracting the negative effect of obesity on bone health.

## Figures and Tables

**Table 1:** Characteristics of the study sample.

	Male *n* = 169	Female *n* = 330	*p* Value
Age (years)	20.53 ± 2.71	20.02 ± 2.79	0.209
Weight (kg)	72.92 ± 11.35	59.74 ± 10.50	0.026
Height (cm)	175.66 ± 7.23	162.04 ± 12.96	0.743
BMI (kg/m^2^)	23.59 ± 3.17	22.51 ± 3.70	0.044
Waist circumference (cm)	83.53 ± 8.14	78.18 ± 8.70	0.820
Body fat %	20.55 ± 6.68	31.93 ± 7.02	0.979
Total body lean mass (kg)	54.11 ± 6.78	37.50 ± 4.52	<0.001
CRF (stages)	8.07 ± 2.13	4.13 ± 1.40	<0.001
Handgrip strength (kg)	40.12 ± 7.23	24.68 ± 4.13	<0.001
Total body BMC (g)	2904.66 ± 482.13	2243.55 ± 279.95	<0.001
Total body BMD (g·cm^−2^)	1.23 ± 0.12	1.12 ± 0.09	<0.001
Calcium(mg/d)	1277.73 ± 1042.99	1179.91 ± 571.67	0.327
Magnesium (mg/day)	499.69 ± 485.61	452.41 ± 230.55	0.290
Phosphorus (mg/day)	2160.89 ± 179.13	1963.65 ± 881.70	0.237
Potassium (mg/day)	5351.02 ± 4955.63	4894.47 ± 2615.83	0.330
Vitamin D (µg/day)	8.64 ± 6.34	7.77 ± 5.99	0.254
Proteins (kcal/day)	510.78 ± 424.64	454.88 ± 209.42	0.157

Values are means ± SDs. Abbreviations: BMI, body mass index; CRF: cardiorespiratory fitness. BMD: bone mineral density; BMC: bone mineral content.

**Table 2:** Partial correlation coefficients between body composition, physical fitness parameters and bone-related nutrients controlling for age and sex.

	Body Fat %	Total Body Lean Mass	CRF	Handgrip	Total Body BMD	Total Body BMC	Calcium	Magnesium	Phosphorus	Potassium	Vitamin D	Proteins
Height	0.046	0.392 **	0.419 **	0.222 **	0.176 **	0.404 **	−0.012	0.019	0.001	0.013	0.010	0.025
Body fat %	-	0.095 *	−0.668 **	−0.460 **	0.157 **	0.128 *	−0.149 **	−0.148 **	−0.146 **	−0.154 **	−0.116	−0.159 *
Total body lean mass		-	0.648 **	0.837 **	0.481 **	0.775 **	−0.006	0.012	0.015	0.024	0.074	0.006
CRF			-	0.633 **	0.330 **	0.483 **	0.129	0.164 *	0.149 *	0.153 *	0.111	0.154 *
Handgrip				-	0.533 **	0.753 **	0.090	0.119	0.108	0.113	0.105	0.116
Total body BMD					-	0.802 **	0.090	0.114	0.122	0.121	0.034	0.038
Total body BMC						-	0.105	0.152	0.153 *	0.156 *	0.080	0.032
Calcium							-	0.881 **	0.921 **	0.857 **	0.477 **	0.855 **
Magnesium								-	0.932 **	0.972 **	0.550 **	0.905 **
Phosphorus									-	0.929 **	0.551 **	0.969 **
Potassium										.	0.541 **	0.897 **
Vitamin D												0.574 **

Abbreviations: CRF: cardiorespiratory fitness. BMD: Bone mineral density; BMC: Bone mineral content. * *p* < 0.05, ** *p* < 0.001.

**Table 3:** ANCOVA models comparing means of body composition and physical fitness variables by ‘Fat But Fit’ categories.

	Cluster (Body Fat %, Handgrip)				Pairwise Comparisons						
	FU	UU	FF	UF	*p* Value	1–2	1–3	1–4	2–3	2–4	3–4
*n*	193	156	72	78							
**Body composition**											
BMI	0.44 ± 1.03	−0.73 ± 0.49	0.51 ± 0.85	−0.14 ± 0.51	<0.001	>		>	<		
Waist circumference	0.43 ± 0.92	−0.71 ± 0.56	0.84 ± 0.89	0.01 ± 0.75	<0.001	>		>	<	<	>
Body fat %	0.81 ± 0.67	−0.34 ± 0.48	−0.45 ± 0.54	−1.51 ± 0.53	<0.001	>		>	˂		>
Total lean mass	−0.19 ± 0.77	−0.53 ± 0.75	1.17 ± 0.60	1.16 ± 0.59	<0.001			<	<	<	
Total body BMC	−0.16 ± 0.79	−0.49 ± 0.80	1.10 ± 0.71	1.00 ± 0.74	<0.001		˂	˂	>	>	
Total body BMD	−0.06 ± 0.88	−0.33 ± 0.89	0.82 ± 0.96	0.66 ± 0.91	0.001		˂	˂	>	>	
**Physical fitness**											
CRF	−0.59 ± 0.59	−0.26 ± 0.75	0.67 ± 0.74	1.32 ± 0.84	<0.001	<		<	>		<
Handgrip strength	−0.41 ± 0.49	−0.61 ± 0.44	1.35 ± 0.73	1.38 ± 0.76	<0.001		<	<	<	<	
**Nutrients**											
Calcium	−0.16 ± 0.70	0.04 ± 0.80	−0.06 ± 0.62	0.53 ± 2.47	0.048			<			
Magnesium	−0.11 ± 0.62	0.02 ± 0.76	−0.08 ± 0.53	0.69 ± 2.73	0.019			<			<
Phosphorus	−0.11 ± 0.70	−0.06 ± 0.75	−0.07 ± 0.49	0.67 ± 2.74	0.026			<			
Potassium	−0.10 ± 0.67	0.04 ± 0.85	−0.07 ± 0.58	0.73 ± 2.74	0.017			<			<
Vitamin D	−0.14 ± 0.82	−0.04 ± 0.94	0.06 ± 0.73	0.17 ± 1.21	0.404						
Proteins	−0.13 ± 0.65	−0.01 ± 0.80	−0.08 ± 0.50	0.72 ± 2.72	0.011			<			<

All variables were normalized. Values are means ± SDs. ANCOVA model were adjusted for height, age and sex. Abbreviations: BMI, body mass index; CRF, cardiorespiratory fitness; FU, Fat Unfit; UU, Unfat Unfit; FF, Fat Fit; UF, Unfat Fit. Symbols: >, < indicate statistical significance (*p* < 0.05) in pairwise mean comparisons using Bonferroni post hoc test.

## Data Availability

The datasets produced during and/or analyzed during the current study are available from the corresponding author (Miriam Garrido-Miguel) on reasonable request.

## Appendix A

**Table A1:** ANCOVA models comparing means of body composition and physical fitness variables by ‘Fat But Fit’ categories (cluster body fat %, CRF)**.**

	Cluster (Body Fat %, CRF)				Pairwise Comparisons						
	FU	UU	FF	UF	*p* Value	1–2	1–3	1–4	2–3	2–4	3–4
*n*	142	208	79	70							
**Body composition**											
BMI	0.68 ± 1.10	−0.56 ± 0.70	0.42 ± 0.69	−0.30 ± 0.49	<0.001	>	>	>	<		>
Waist circumference	0.71 ± 1.01	−0.48 ± 0.56	0.64 ± 0.89	−0.30 ± 0.54	<0.001	>	>	>	<	<	>
Body fat %	0.96 ± 0.56	−0.01 ± 0.47	−0.56 ± 0.83	−1.37 ± 0.54	<0.001	>	>	>	˂	>	>
Total lean mass	−0.13 ± 0.79	−0.45 ± 0.59	1.33 ± 0.90	1.17 ± 0.88	<0.001	<	<	<	<	<	
Total body BMC	−0.09 ± 0.89	−0.40 ± 0.68	1.13 ± 1.19	0.91 ± 1.00	<0.001	>	˂	˂	>	>	
Total body BMD	−0.02 ± 0.96	−0.34 ± 0.78	0.82 ± 1.21	0.61 ± 0.97	0.001	>	˂	˂	>	>	
**Physical fitness**											
CRF	−0.75 ± 0.42	−0.36 ± 0.47	1.08 ± 0.63	1.59 ± 0.61	<0.001	<		<	>		<
Handgrip strength	−0.30 ± 0.76	−0.42 ± 0.65	1.13 ± 0.87	0.95 ± 0.89	<0.001		<	<	<	<	
**Nutrients**											
Calcium	−0.23 ± 0.63	−0.04 ± 0.74	−0.02 ± 0.55	0.13 ± 0.83	0.048			<			
Magnesium	−0.17 ± 0.52	−0.03 ± 0.74	−0.02 ± 0.50	0.29 ± 1.02	0.030			<			
Phosphorus	−0.19 ± 0.52	−0.05 ± 0.78	−0.03 ± 0.47	0.27 ± 0.99	0.022			<			
Potassium	−0.17 ± 0.56	−0.02 ± 0.80	−0.03 ± 0.56	0.38 ± 1.10	0.023			<			
Vitamin D	−0.19 ± 0.80	−0.02 ± 0.96	0.09 ± 0.67	0.02 ± 0.87	0.401						
Proteins	−0.20 ± 0.49	−0.04 ± 0.83	−0.07 ± 0.46	0.36 ± 1.10	0.012			<		<	

All variables were normalized. Values are means ± SDs. ANCOVA model were adjusted for height, age and sex. Abbreviations: BMI, body mass index; CRF, cardiorespiratory fitness; FU, Fat Unfit; UU, Unfat Unfit; FF, Fat Fit; UF, Unfat Fit. Symbols: >, < indicate statistical significance (*p* < 0.05) in pairwise mean comparisons using Bonferroni post hoc test.
